# *Escherichia coli* AraJ boosts utilization of arabinose in metabolically engineered cyanobacterium *Synechocystis* sp. PCC 6803

**DOI:** 10.1186/s13568-021-01277-7

**Published:** 2021-08-13

**Authors:** Saurabh Ranade, Qingfang He

**Affiliations:** Department of Biology, University of Arkansas at Little Rock, 2801 South University Avenue, Little Rock, AR 72204 USA

**Keywords:** Cyanobacteria, Metabolic engineering, *Synechocystis*, Arabinose transporter, AraJ, AraBAD

## Abstract

Lignocellulosic biomass can serve as an inexpensive and renewable source of carbon for the biosynthesis of commercially important compounds. L-arabinose is the second most abundant pentose sugar present in the plant materials. Model cyanobacterium *Synechocystis* sp. PCC 6803 is incapable of catabolism of L-arabinose as a source of carbon and energy. In this study, all the heterologous genes expressed in *Synechocystis* were derived from *Escherichia coli* K-12. Initially we constructed four *Synechocystis* strains that expressed AraBAD enzymes involved in L-arabinose catabolism, either in combination with or without one of the three arabinose transporters, AraE, AraFGH or AraJ. Among the recombinants, the strain possessing AraJ transporter was observed to be the most efficient in terms of dry biomass production and L-arabinose consumption. Later, an additional strain was generated by the expression of AraJ in the AraE-possessing strain. The resultant strain was shown to be advantageous over its parent. This study demonstrates that AraJ, a protein with hitherto unknown function plays a role in the uptake of L-arabinose to boost its catabolism in the transgenic *Synechocystis* strains. The work also contributes to the current knowledge regarding metabolic engineering of cyanobacteria for the utilization of pentose sugars.

**Supplementary Information:**

The online version contains supplementary material available at 10.1186/s13568-021-01277-7.

## Key points

*Synechocystis* strains were engineered to transport and catabolize L-arabinose.

L-arabinose transport took place even in the absence of heterologous transporters.

Expression of putative transporter gene *araJ* showed the most impressive results.

## Introduction

**Fig. 1:**
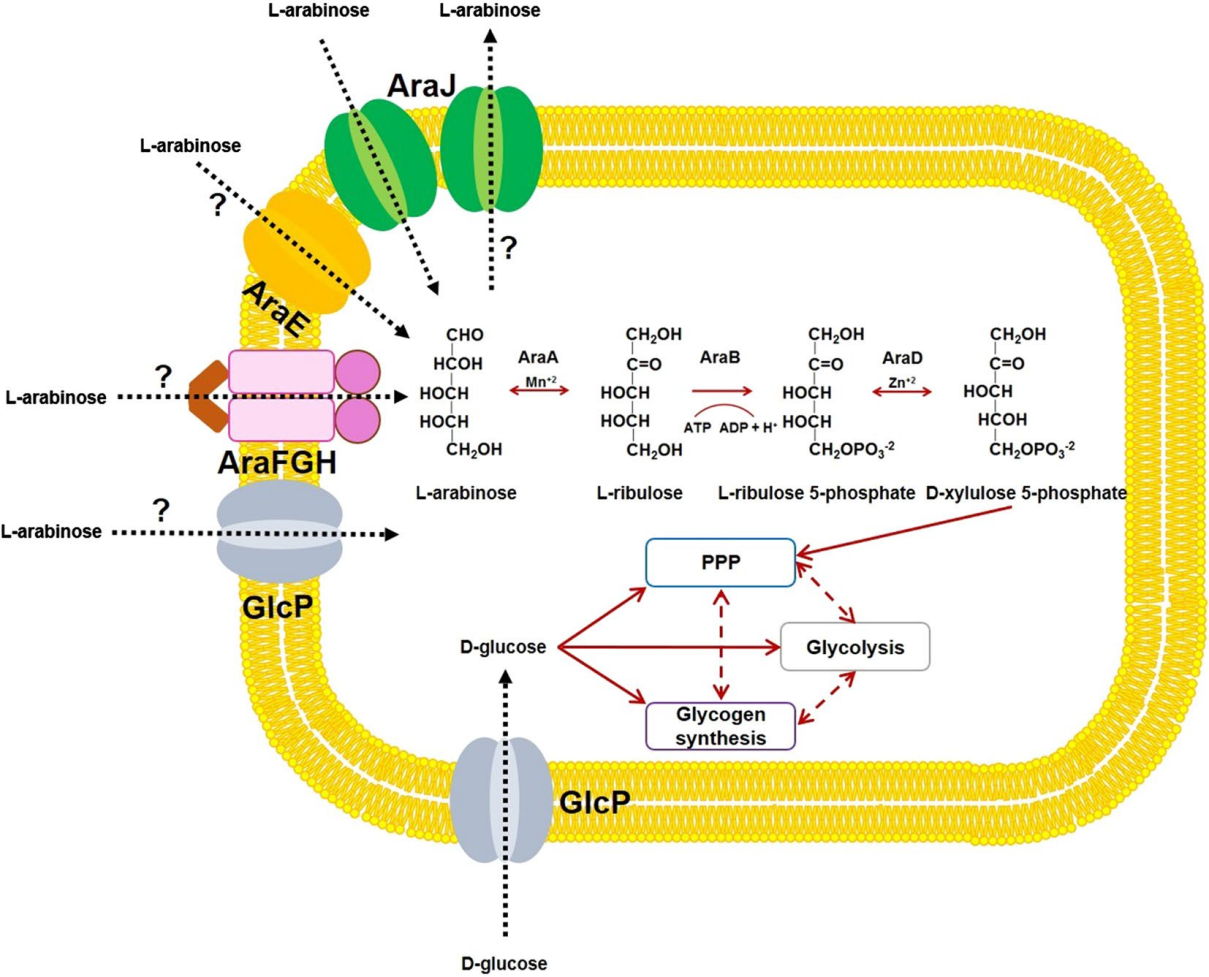
Metabolic scheme for engineering *Synechocystis* strains. L-arabinose may enter the transformant strains by means of native transporter/s such as GlcP and heterologous transporters such as AraE, AraFGH and AraJ. Direction of entry and exit of sugars (established, indicated in this study and putative) is shown using dotted arrows. L-arabinose is converted to L-ribulose by L-arabinose isomerase (AraA) in the presence of manganese ions (Mn^+2^). L-ribulose is phosphorylated to L-ribulose 5-phosphate (L-Ru5P) by the action of L-ribulokinase (AraB). L-Ru5P is converted by L-ribulose 5-phosphate 4-epimerase (AraD) in the presence of zinc ions (Zn^+2^) to D-xylulose 5-phosphate (D-Xu5P), which enters the pentose phosphate pathway (PPP). Bidirectional arrows indicate a reversible reaction or connections between metabolic pathways through intermediates. Unidirectional arrows indicate a non-reversible reaction or the ability of a sugar or an intermediate to either enter or exit a pathway. Dashed arrows indicate the involvement of one or more intermediates. Question marks (?) indicate putative transport of L-arabinose. Other abbreviations: ADP, adenosine diphosphate; ATP, adenosine triphosphate

Biomass is an abundant and renewable raw material that can be converted into a number of useful products, transportation fuels and direct energy (Ragauskas et al. 2006). Hemicelluloses which comprise of heterogeneous polymers of pentoses, hexoses, and organic acids represent about 20–35% of the total lignocellulosic biomass (Saha 2003). Xylose stands out as the predominant pentose sugar in the hardwood hemicelluloses. However, L-arabinose is one of the major pentose sugars in the leaves of grass and other herbaceous species, and also in agricultural residues such as rapeseed meal, corn fibre, barley straw, rice straw, wheat bran etc. (Mohagheghi et al. 2002; Schädel et al. 2010; Gìrio et al. 2010; Lomascolo et al. 2012). In arabinose-utilizing bacteria, L-arabinose is transported across the cell membrane by means of specialized transporter proteins. In the first step after transportation, L-arabinose is isomerized to L-ribulose by the action of AraA (L-arabinose isomerase). L-ribulose is irreversibly phosphorylated by AraB (L-ribulokinase) to form L-ribulose-5-phosphate (hereafter L-Ru5P). In the last step, L-Ru5P is converted by AraD (L-ribulose-5-phosphate 4-epimerase) to a pentose phosphate pathway intermediate, D-xylulose-5-phosphate (hereafter D-Xu5P) (Schleif 2010; Caspi et al. 2018) (Fig. 1).

Unlike glucose, pentose sugars are less susceptible to bacterial action. Among the few bacterial species that possess the natural ability to utilize pentose sugars, productivity and yield remain low owing to a variety of issues such as insufficient transport, catabolite repression, and cellular redox imbalance in pentose metabolism. Hence, transformation of industrial strains with heterologous pentose pathway-specific genes has been a popular strategy to enable the strains to grow on lignocellulosic hydrolysates (Jojima et al. 2010). Cyanobacterium, *Synechocystis* sp. PCC 6803 (hereafter *Synechocystis*) is one of the most extensively studied primary phototrophs. It is able to grow under autotrophic, mixotrophic, and heterotrophic conditions, can integrate foreign DNA at a high frequency, and holds a proven record as a host strain for the synthesis of biofuels, health related compounds, and commodity chemicals (Yu et al. 2013).

Several groups have engineered bacterial strains, either to enable them to utilize L-arabinose or to improve their efficiency to utilize the wood sugar. In an early study, arabinose utilization genes *araBAD* and pentose phosphate pathway genes *talB*-*tktA* from *Escherichia coli* were introduced into *Zymomonas mobilis* ATCC 39676 under the control of GAP and ENO promoters, respectively, in order to obtain a co-fermenting strain which was able to convert L-arabinose into ethanol at 98% maximum theoretical yield (Deanda et al. 1996). In the study by Mohagheghi et al. (2002), *E*. *coli* genes involved in arabinose, xylose, and pentose phosphate pathway were stably integrated into the genome of *Z*. *mobilis* to obtain a strain which was able co-ferment xylose, arabinose, and glucose into ethanol at 84% process yield. Similarly, *E*. *coli araBAD* genes were expressed under *lac* promoter in *Corynebacterium glutamicum* R to generate a strain which was able to produce organic acids from L-arabinose even in the presence of glucose (Kawaguchi et al. 2008). The work by Kawaguchi et al. (2008) was further enhanced by the introduction of *trc* promoter-controlled pentose transporter gene *araE* from *C*. *glutamicum* ATCC 31831 to the parental strain harbouring *araBAD* genes. The resultant strain showed a 2.8-fold increase in the L-arabinose consumption rates under oxygen-deprived conditions. The strain could also grow aerobically on L-arabinose at lower concentrations in comparison with the parental strain (Sasaki et al. 2009). In one of the first reported works involving cyanobacteria, Cao et al. (2017) constructed an L-arabinose-based inducible module comprising of *E*. *coli araBAD* promoter in *Synechococcus elongatus* PCC 7942. Expression of *E*. *coli araE* under *trc* promoter failed to improve arabinose transport. To alleviate inhibitory effects of L-arabinose in the cells, *E*. *coli araBAD* genes were expressed under J23119, a constitutive promoter. After 10 days of cultivation, the consumption of L-arabinose was estimated at 0.40 g/L in diurnal and 0.84 g/L in continuous light condition, where the corresponding O.D_730_ values were ~ 1.3 and ~ 2.0, respectively. In addition to the bacterial strains, several arabinose-utilizing yeast strains have been generated by the introduction of transporter and catabolic genes of bacterial or fungal origin (Young et al. 2010).

In this study, we compared the biomass yield and L-arabinose uptake of four recombinant *Synechocystis* strains heterologously expressing arabinose-specific catabolic genes *araBAD* without or with one of three arabinose transporters (AraE, AraFGH, AraJ) under consideration. AraE (proton symporter) and AraFGH are well characterized members of the Major Facilitator Superfamily (hereafter MFS) class and ATP Binding Cassette (hereafter ABC) class of transporter proteins, respectively (Jojima et al. 2010). In *E*. *coli*, the exact function of AraJ has remained unknown (Reeder and Schleif 1991; Fritz et al. 2014). However, in terms of homology, AraJ has been grouped with multidrug efflux proteins of the MFS, as a member of drug H^+^ antiporter (DHA1) family (Bost et al. 1999; Kanehisa et al. 2016). All the arabinose-specific genes used in this study were sourced from *E*. *coli* K-12. We further showed that the expression of AraJ in the strain possessing AraE improved arabinose consumption and alleviated stress. Earlier, we had constructed *Synechocystis* strains with varying abilities to utilize D-xylose and showed that xylose consumption could be enhanced by the expression of efficient transporters (Ranade et al. 2015). Here, we report the generation of recombinant *Synechocystis* strains with various abilities to utilize L-arabinose. Our data suggests that AraJ, a poorly characterized MFS protein, is involved in the uptake of L-arabinose when expressed in *Synechocystis*.

## Materials and methods

### Bacterial strains and growth conditions

*E. coli* strain K-12 was used as the source organism for amplification of arabinose-specific transporter and catabolic genes, while *E. coli* TOP10 (Thermo Fisher Scientific) was used for cloning and plasmid construction steps. The strains were grown at 37 °C on solid LB medium or in liquid LB medium with shaking (220 rpm) in the presence of appropriate antibiotic, if necessary. Antibiotic concentrations used for selection were- ampicillin: 100 µg/mL, kanamycin: 25 µg/mL, erythromycin: 200 µg/mL, and spectinomycin: 50 µg/mL. The antibiotics were purchased from Thermo Fisher Scientific.

### Plasmid construction

To construct the transformation vectors designed to insert arabinose transporter genes into *Synechocystis* wild-type strain (hereafter WT), kanamycin resistance cassette, upstream and downstream regions of a neutral site (near *slr1285*, denoted as neutral site 1), a ~ 0.4 kb region encompassing the *psbA2* promoter (denoted as *psbA2* promoter) and a ~ 0.5 kb 5ST1T2 double terminator region were inserted into pBluescript II SK+ (Stratagene) as described (Ranade et al. 2015). The arabinose transporter genes *araE*, *araFGH*, and *araJ* were amplified from genomic DNA isolated from *E. coli* K-12 strain. The primers used to amplify the transporter genes were as follows:

5′-ACTCGAATTCATGGTTACTATCAATACGGAATC-3′ (forward primer) and 5′-ACTCCTGCAGTCAGACGCCGATATTTCTC-3′ (reverse primer) for *araE*;

5′-ACTCGAATTCATGCACAAATTTACTAAAGCCCTG-3′ (forward primer) and 5′-ACTCCTGCAGTCAGACAGTGCGTTTCGCTTTTTG-3′ (reverse primer) for *araFGH*;

5′-ACTCGAATTCATGAAAAAAGTCATTTTATCTTTGGCTC-3′ (forward primer) and 5′-ACTCCTGCAGCTACCCCAGTGGTTTCGCCAG-3′ (reverse primer) for *araJ*.

The amplicons were inserted between *Eco*RI-*Pst*I sites of the plasmid to generate three individual plasmids. Note that, in the case of *araFGH* gene set, only the region from the start codon of *araF* to the end codon of *araG* was amplified; no other regulatory elements from the *E*. *coli araFGH* operon were included.

To construct the plasmid designed to insert arabinose-specific catabolic genes into *Synechocystis* strains possessing exogenous arabinose transporters and in the WT strain, erythromycin resistance cassette was amplified from plasmid pTSC (Yan et al. 2008) using the following primers:

5′-ACTCGGATCCGAGCTCGTGCTATAATTATACTAATTTTATAAG-3′ (forward primer) and 5′-ACTCGGATCCATCGATTCACAAAAAATAGGCACACG-3′ (reverse primer). The amplicon was inserted into pBluescript II SK+ plasmid at *Bam*HI site. The neutral site sequence (near *slr0646*; hereafter referred to as neutral site 2) (Gonzalez-Esquer and Vermaas 2013) was amplified from genomic DNA isolated from *Synechocystis* as upstream and downstream regions using the following primers:

5′-ACTCGGTACCTTCTGTAAGCACTTCGATCGTTAG-3′ (forward primer) and 5′-ACTCCTCGACATGGGGATCAGCGCTAAATGC-3′ (reverse primer) for the upstream region, and 5′-ACTCACTAGTAAGCCCATTTACGTCGTGTTGGTC-3′ (forward primer) and 5′-ACTCCCGCGGCCAACGGTTCCAGGTGACTATC-3′ (reverse primer) for the downstream region. The upstream and downstream fragments were inserted between *Kpn*I-*Xho*I and *Spe*I-*Sac*II sites of the plasmid, respectively. The *psbA2* promoter and 5ST1T2 double terminator were amplified and cloned into the plasmid as described (Ranade et al. 2015). In the last step, the arabinose catabolic gene set *araBAD* was amplified from genomic DNA isolated from the *E. coli* K-12 strain using the following primers:

5′-ACTCGAATTCATGGCGATTGCAATTGGCCTC-3′ (forward primer) and

5′-ACTCCCTGCAGGTTACTGCCCGTAATATGCCTTC-3′ (reverse primer). The amplicon was digested at the *Eco*RI-*Sbf*I restriction sites present at its ends and inserted between the *Eco*RI-*Pst*I sites of the plasmid. Similar to *araFGH*, in the case of *araBAD* gene set, only the region from the start codon of *araB* to the end codon of *araD* was amplified; no other regulatory elements from the *E*. *coli araBAD* operon were included.

To construct the plasmid vector for the insertion of *araJ* into the engineered strain possessing *araE* and *araBAD*, spectinomycin resistance cassette, upstream and downstream regions of a neutral site (*slr1608*, denoted as neutral site 3), *psbA2* promoter and 5ST1T2 double terminator region were inserted into pBluescript II SK+ (Stratagene) as described (Ranade et al. 2015). In the last step, *araJ* was amplified from *E. coli* K-12 genomic DNA using the primer pair described above, and was inserted between *Eco*RI-*Pst*I sites of the plasmid.

### Transformation and segregation of Synechocystis strains

*Synechocystis* strains were grown at 30 °C on solid BG-11 and in liquid BG-11 medium at 50–60 µE m^-2^s^-1^ light intensity, with constant shaking (200 rpm). When OD_730_ reached ~ 0.7–0.8, the transformations were carried out as described (Porter 1988). Upon confirmation of the presence of heterologous genes by PCR analyses, cells were streaked on BG-11 agar plates containing successively higher antibiotic concentrations. PCR checks were performed to ensure complete segregation of the transformants.

### Reverse transcription PCR

Total RNA was isolated from cultures of *Synechocystis* strains at OD_730_ ~ 0.7 as previously described (Mohamed and Jansson 1989). Turbo DNA-free Kit (Thermo Fisher Scientific) was used to remove carried over genomic DNA per manufacturer’s instructions. The reverse transcription reactions were carried out using Superscript III enzyme (Life Technologies) and random hexamers (New England BioLabs). The cDNA molecules were then used as templates for PCR, employing the same set of primers used to amplify the heterologous genes. As negative controls, DNase-treated RNA molecules were used as templates, and the same primer pairs were employed. *petA* (*sll1317*) was used as the positive control as described (Ranade et al. 2015). For longer templates such as *araFGH* and *araBAD* gene sets, the SuperScript One-Step RT-PCR system for long templates (Thermo Fisher Scientific) was used per manufacturer’s instructions. PCR products were analyzed on 0.8% agarose gel.

### Biomass measurement

Growth exhibited by *Synechocystis* strains was measured in terms DW as described (Davies et al. 2014). Biomass estimation studies were performed concurrently with the enzymatic assays. The cultures of *Synechocystis* strains were initiated in 110 mL BG-11 liquid medium at 30 °C under shaking conditions (200 rpm), with the initial OD_730_ of 0.05. The cultures were provided with: for autotrophy, ~ 60 μE m^-2^ s^-1^ light; for mixotrophy, 20 mM L-arabinose (3.00 g/L) or 20 mM D-glucose (3.60 g/L) or 10 mM each of L-arabinose (1.50 g/L) and D-glucose (1.80 g/L) along with ~ 60 μE m^-2^ s^-1^ light. Biomass values were estimated at 6-h intervals for the first 3 days followed by 24-h intervals for the next 4 days.

### Enzymatic assays for measurement of sugar uptake

Enzymatic uptake assays were performed for the cultures grown only under mixotrophy. During the assays, 1 mL cultures were collected at the same time intervals specified for biomass measurements. The cultures were immediately filtered using 0.45 µm pore size 13 mm nylon membrane syringe filters (Fisher Scientific) to obtain cell-free media.

The amount of L-arabinose present in the filtered media was measured using L-Arabinose/D-Galactose Assay Kit (Megazyme) per manufacturer’s instructions. Sequential action of galactose mutarotase and β-galactose dehydrogenase generates NADH molecules. The amount of NADH is stoichiometric with that of L-arabinose which was calculated from the difference in OD_340_ values before and after the β-galactose dehydrogenase action.

The amount of D-glucose present in the filtered media was measured using D-Glucose Assay Kit-GOPOD Format (Megazyme) per manufacturer’s instructions. Action of glucose oxidase and peroxidase generates quinoneimine dye. The amount of quinoneimine formed is stoichiometric with that of D-glucose. The amount of D-glucose in the cell-free media was calculated from OD_510_ values obtained for the samples, in reference to the OD_510_ value read for a glucose standard.

## Results

### Comparative performance studies of Synechocystis strains

#### Construction of recombinant strains of *Synechocystis*

For the purpose of comparative performance studies, *Synechocystis* strains possessing arabinose transporter and catabolic genes were developed from the WT strain in two distinct rounds of transformation by homologous double recombination. In the first round, three recombinant strains possessing one of the three transporter genes, *araE* or *araFGH* or *araJ* of *E*. *coli* K-12 origin were generated. The strains were named A-Tr1, A-Tr2 and A-Tr3, respectively, and collectively referred to as A-Tr. In the subsequent round of transformation, four recombinant strains were generated by inserting the *araBAD* gene set of *E*. *coli* K-12 origin into the three strains generated in the first round of transformation and the WT strain. The recombinant strains were named A-Ut1, A-Ut2, A-Ut3 and A-Ut4, respectively, and collectively referred to as A-Ut (Table 1). In this study, *araFGH* and *araBAD* will hereafter be referred to as *araFGH* and *araBAD* gene sets, respectively. *araFGH* gene set includes the region from the start codon of *araF* gene to the end codon of *araH* gene. Similarly, *araBAD* gene set includes the region from the start codon of *araB* gene to the end codon of *araD* gene.

**Table 1:** *Synechocystis* strains used in this study

Strain	Genotype	Description
WT	Wild-type *Synechocystis* sp. 6803	Wild-type genomic sequence
A-Tr1	*araE*-Δ*Neu 1*	*araE* inserted at neutral site 1 (near *slr1285*)
A-Tr2	*araFGH*-Δ*Neu 1*	*araFGH* gene set inserted at neutral site 1 (near *slr1285*)
A-Tr3	*araJ*-Δ*Neu 1*	*araJ* inserted at neutral site 1 (near *slr1285*)
A-Ut1	*araE*-Δ*Neu 1*::*araBAD*-Δ*Neu 2*	*araE* inserted at neutral site 1 (near *slr1285*), *araBAD* gene set inserted at neutral site 2 (near *slr0646*)
A-Ut2	*araFGH*-Δ*Neu 1*::*araBAD*-Δ*Neu 2*	*araFGH* gene set inserted at neutral site 1 (near *slr1285*), *araBAD* gene set inserted at neutral site 2 (near *slr0646*)
A-Ut3	*araJ*-Δ*Neu 1*::*araBAD*-Δ*Neu 2*	*araJ* inserted at neutral site 1 (near *slr1285*), *araBAD* gene set inserted at neutral site 2 (near *slr0646*)
A-Ut4	*araBAD*-Δ*Neu 2*	*araBAD* gene set inserted at neutral site 2 (near *slr0646*)
A-Ut5	*araE*-Δ*Neu 1*::*araBAD*-Δ*Neu 2*::*araJ*-Δ*Neu 3*	*araE* inserted at neutral site 1 (near *slr1285*), *araBAD* gene set inserted at neutral site 2 (near *slr0646*), *araJ* inserted at neutral site 3 (*slr1608*)

#### Verification of genetic transformation and segregation of the recombinants

The heterologous genes were introduced into the neutral sites of *Synechocystis* genome by homologous recombination. After each round of transformation, the presence of heterologous gene insert was verified. Upon verification of transformation, the recombinant strains were segregated in the presence of increasing antibiotic pressure. The segregated strains were used in the next round of transformation and for further work.

**Fig. 2:**
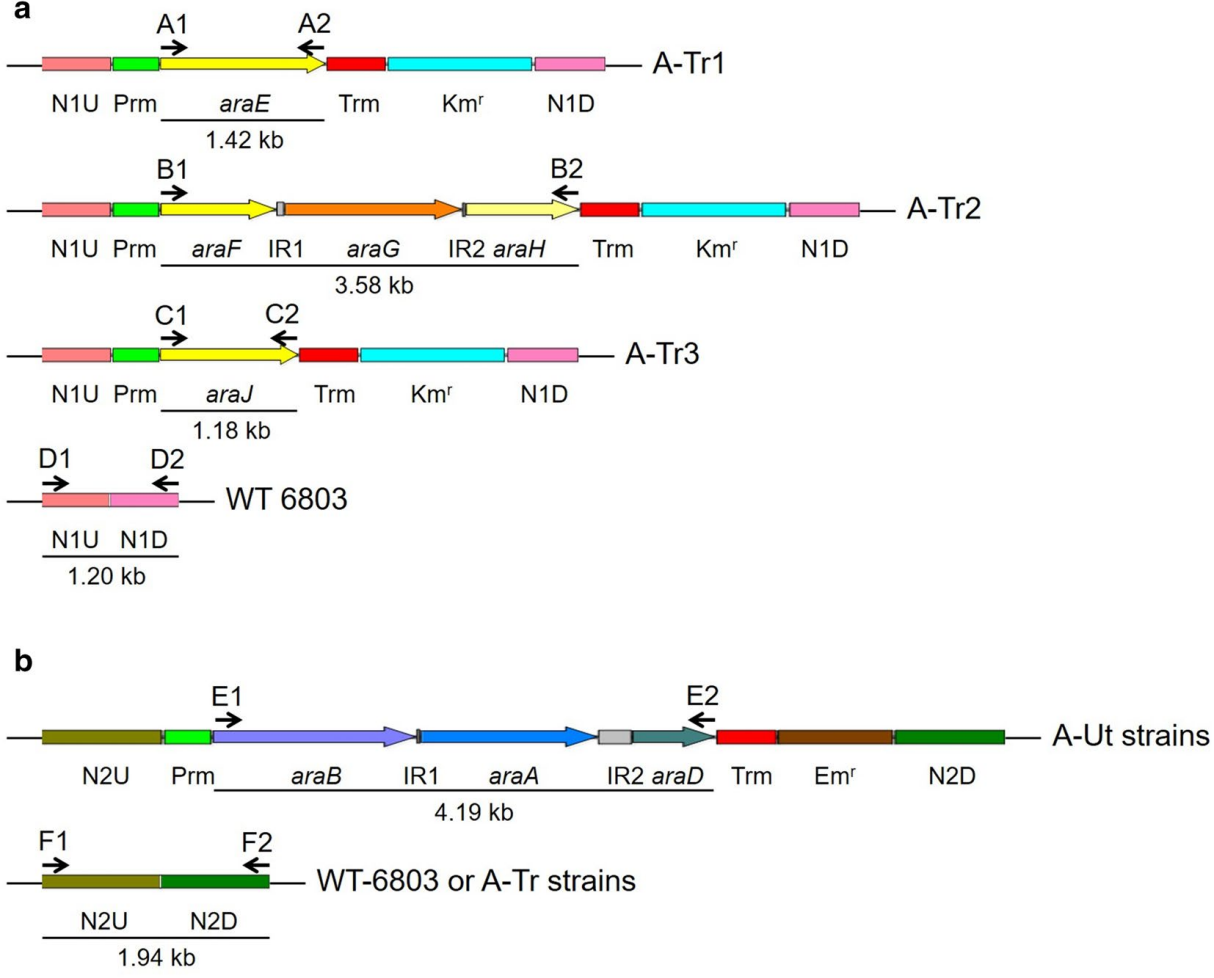
Transformation of *Synechocystis* strains. All transgenes used in the study were expressed under the control of the *psbA2* promoter (Prm) and 5ST1T2 terminator (Trm). Intergenic regions (IR) present between individual genes in the gene sets are represented as grey boxes. Numbered letters and arrows represent the primer pairs, and the direction of the amplifications, respectively. **a** Genes encoding the arabinose transporters were inserted into neutral site 1 in the *Synechocystis* WT genome using upstream (N1UP) and downstream (N1DW) regions for homologous recombination. The kanamycin resistance cassette (Km^r^) was used for the selection and segregation of the transformants. **b** The *araBAD* gene set, encoding enzymes that funnel arabinose into the pentose phosphate pathway, was inserted into neutral site 2 in the genomes of *Synechocystis* strains carrying one of the three arabinose transporter genes and the WT strain using upstream (N2UP) and downstream (N2DW) regions for homologous recombination. The erythromycin resistance cassette (Em^r^) was used for the selection and segregation of the transformants

**Fig. 3:**
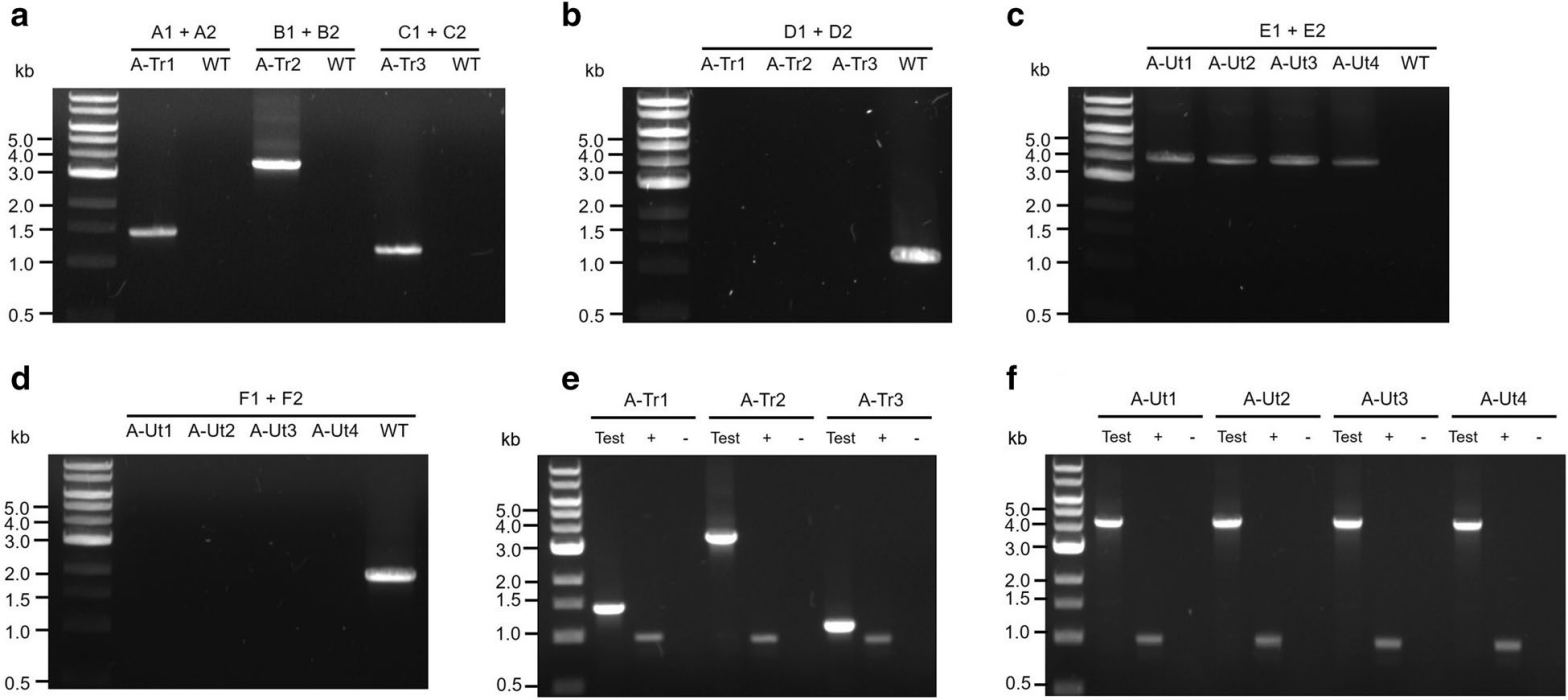
Evaluation of transformation, chromosomal segregation and transcription. **a** The presence of transporter genes was verified with the help of primers A1-A2 (*araE*), B1-B2 (*araFGH*) and C1-C2 (*araJ*). The primers produced fragments of ~1.42, ~3.58 and ~1.18 kb, respectively. WT genomic DNA served as the negative control. **b** Chromosomal segregation was examined with the help of primers D1-D2. In the absence of uninterrupted neutral site 1, the primers failed to generate PCR products, whereas the WT genomic DNA serving as the positive control yielded a ~1.20 kb product. **c** The presence of *araBAD* gene set was verified with the help of primers E1-E2. The primers produced ~4.19 kb band. WT genomic DNA was used as the negative control. **d** Chromosomal segregation was verified with the help of primers F1-F2. In the absence of uninterrupted neutral site 2, the primers failed to generate any PCR product, whereas the WT genomic DNA included as the positive control yielded a ~1.94-kb product. (e) Transcription of the transporter genes was demonstrated with the help of primers A1-A2 (*araE*), B1-B2 (*araFGH*) and C1-C2 (*araJ*), represented as ‘Test’. The primers generated products of ~1.42, ~3.58 and ~1.18 kb, respectively. As the positive controls (+), transcription of *petA* (~0.99-kb) was examined in the cDNA templates. As the negative controls (-), amplification of the transporter genes from the corresponding RNA samples was evaluated. (f) Transcription of *araBAD* gene set was demonstrated with the help of primers E1-E2, represented as ‘Test’. The primers generated products of ~4.19 kb. As the positive controls (+), transcription of *petA* (~0.99-kb) was examined in the cDNA templates. As the negative controls (-), amplification of the catabolic gene set from the corresponding RNA samples was evaluated

To verify the presence of heterologous transporter genes in the strains generated after the first round of transformation, i.e., A-Tr1, A-Tr2 and A-Tr3, the same pairs of primers used to amplify the respective genes from *E*. *coli* K-12 genomic DNA were used. Primers A1/A2 for *araE*, B1/B2 for *araFGH* and C1/C2 for *araJ* generated products of approximately 1.42 kb, 3.58 kb and 1.18 kb in length, respectively (Figs. 2a and 3a). To verify segregation of the recombinants, the forward primer used to amplify the upstream region and the reverse primer used to amplify the downstream region of neutral site 1 were used (D1/D2) (Fig. 2a). Extension times in the PCR cycles were set specifically to amplify the uninterrupted neutral site 1. PCR amplifications produced a ~ 1.20 kb band representing neutral site 1 only in the WT strain maintained as the positive control. The results showed the absence of uninterrupted neutral site 1 and hence complete segregation of the A-Tr strains (Fig. 3b).

To test for the presence of catabolic gene set *araBAD* in the strains obtained after the second round of transformation, i.e., A-Ut1, A-Ut2, A-Ut3 and A-Ut4, the same pair of primers used to amplify *araBAD* from *E*. *coli* K-12 genomic DNA was used. Primers E1/E2 for *araBAD* gene set generated products of approximately 4.19 kb in length (Figs. 2b and 3c). To verify segregation, the forward primer used to amplify the upstream region and the reverse primer used to amplify the downstream region of neutral site 2 were used (F1/F2) (Fig. 2b). Extension times in the PCRs were set to amplify the uninterrupted neutral site 2. PCR amplifications produced a ~ 1.94 kb band representing neutral site 2 only in the WT strain maintained as the positive control. The results indicated the absence of uninterrupted neutral site 2 and hence complete segregation of the A-Ut strains (Fig. 3d).

#### Verification of transcription of heterologous genes

After verification of segregation, transcription of the arabinose-specific heterologous genes in the A-Tr and A-Ut strains was examined by RT-PCR. As the positive control, expression of *petA* (*sll1317*) coding for apocytochrome *f*, a core subunit of cytochrome *b*_*6*_*f* complex was tested. As the negative control, DNase-treated RNA samples obtained from each strain were tested to ensure the absence of any leftover DNA in the RNA samples. In the presence of cDNA templates obtained from the respective strains, primers A1/A2 for *araE*, B1/B2 for *araFGH*, C1/C2 for *araJ* and E1/E2 for *araBAD* generated products of expected sizes. In negative control reactions, the aforementioned primer pairs failed to generate any product. A ~0.98-kb band representing *petA* was obtained for positive controls in all the transformants (Figs. 2a, b and 3e, f). The results indicate successful transcription of all the heterologous genes/ gene sets and verify the quality of the RNA samples.

#### Study of biomass accumulation by the recombinants

To study and compare the growth patterns exhibited by the WT, A-Tr and A-Ut strains, they were cultured under various conditions. For biomass measurements, cells were collected at 6-h intervals for the first 3 days and then at 24-h intervals for the next 4 days. Under autotrophy, biomass yields were similar for all the strains. Under mixotrophy with 20 mM glucose (hereafter mixotrophy-glucose), biomass yields were observed to be similar for all the strains except for the AraJ-possessing A-Tr3 and A-Ut3 strains. A-Tr3 and A-Ut3 strains showed a degree of growth impairment in the presence of 20 mM glucose (Additional File 1: Figure S1a–h).

**Fig. 4:**
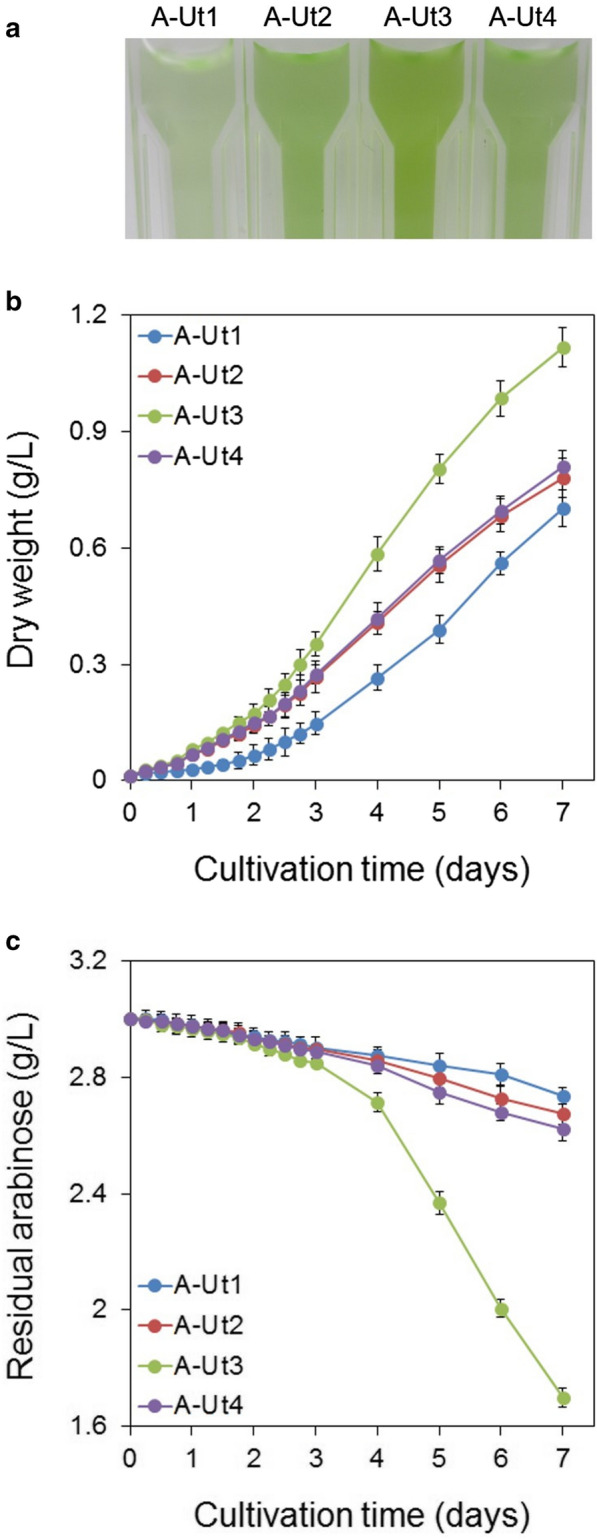
Growth and arabinose consumption under mixotrophy in the presence of 20 mM arabinose. *Synechocystis* strains A-Ut1, A-Ut2, A-Ut3 and A-Ut4 were grown in the presence of 20 mM (3.0026 g/L) L-arabinose. **a** Photograph depicting differential growth shown by the transformant strains at the end of the fourth day of culture. **b** Dry biomass and **c** Residual arabinose measured in the cultures of the transformant strains. Data represented in graphs were collected from three biological replicates and presented as means ± standard deviations

Under mixotrophy with 20 mM arabinose (hereafter mixotrophy-arabinose), the WT and the A-Tr strains showed biomass yields similar to those obtained under autotrophy, indicating the inability of the strains to utilize arabinose. However, the A-Ut strains showed greater dry weight (hereafter DW) yields under the mixotrophy-arabinose condition than under autotrophy. Biomass accumulation results demonstrate that all the A-Ut strains carrying the catabolic gene set were able to utilize arabinose. Under the mixotrophy-arabinose condition, at the 7th day of culture, the biomass reached by the A-Ut1, A-Ut2, A-Ut3 and A-Ut4 strains was ~ 31%, 43%, 112%, and ~ 50% greater than the average biomass for the four strains under autotrophy. In the presence of arabinose, AraJ-possessing A-Ut3 strain showed higher biomass values than the other A-Ut strains at each time point. At the 7th day of culture, A-Ut3 showed ~ 38% greater biomass than the A-Ut4 strain, which lacked any heterologous transporter (Fig. 4a, b; Additional File 1: Figure S1b, d, f, h).

**Fig. 5:**
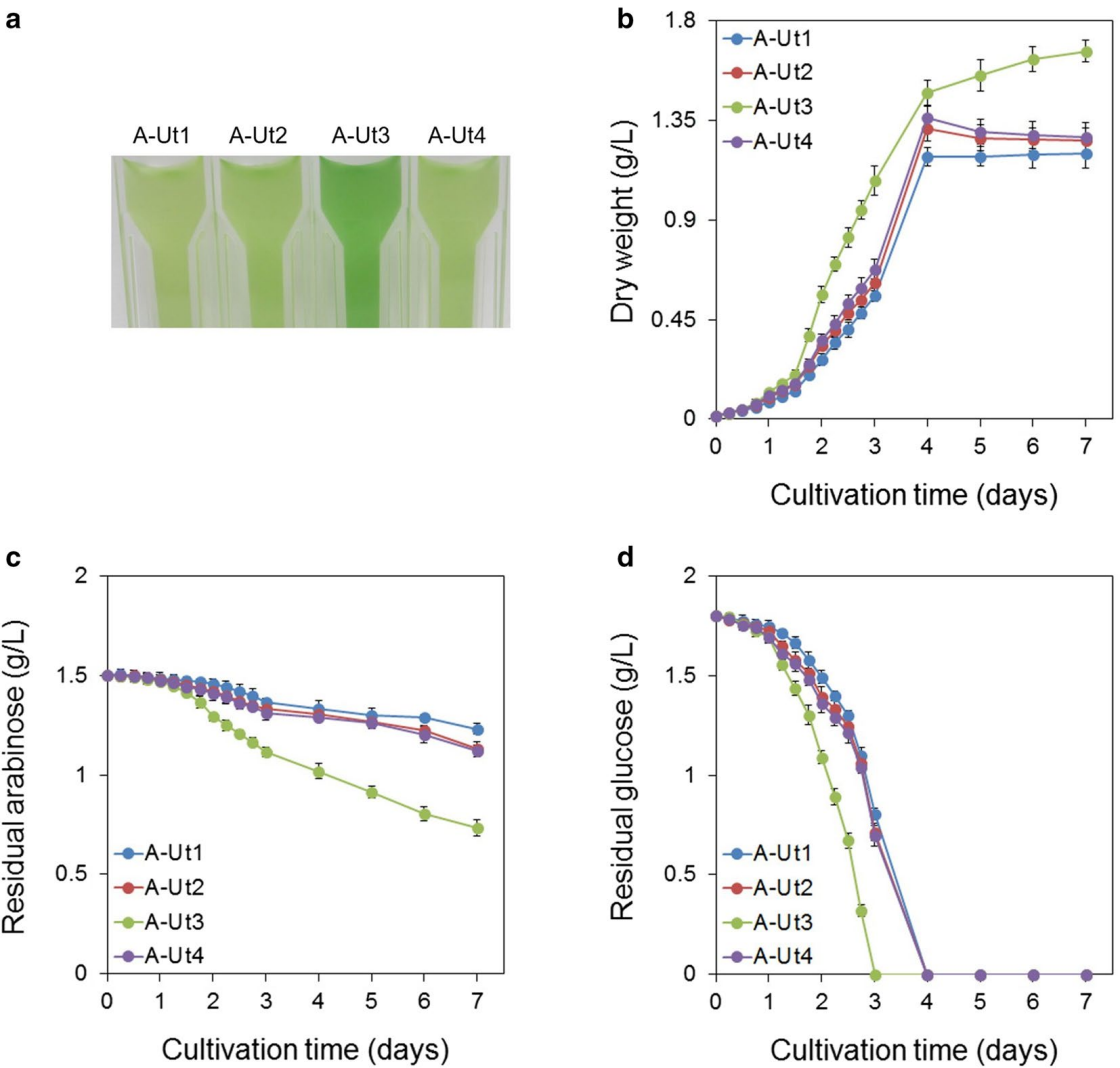
Growth and sugar consumption in the presence of 10 mM arabinose and glucose. *Synechocystis* strains A-Ut1, A-Ut2, A-Ut3 and A-Ut4 were grown in the presence of 10 mM (1.50 g/L) L-arabinose and 10 mM (1.80 g/L) D-glucose. **a** Photograph depicting differential growth shown by the transformant strains at the end of the third day of culture. **b** Dry biomass, **c** Residual arabinose and **d** Residual glucose measured in the cultures of the transformant strains. Data represented in graphs were collected from three biological replicates and presented as means ± standard deviations

To further examine the ability of the arabinose-consuming A-Ut strains to grow in the presence of arabinose and glucose, we cultured the strains under mixotrophy, in the presence of 10 mM each of arabinose and glucose (hereafter mixotrophy-mixed sugar). Under the mixotrophy-mixed sugar condition, at the 7th day of culture, the biomass reached by the arabinose-consuming strains, A-Ut1, A-Ut2, A-Ut3 and A-Ut4 was ~ 124%, 130%, 215%, and ~ 136% greater than the average biomass for the four strains accumulated under autotrophy. Similar to the mixotrophy-arabinose condition, the A-Ut3 strain showed higher biomass values than the other A-Ut strains at each time point tested. At the end of the last day of culture, A-Ut3 showed ~31% greater biomass than the A-Ut4 strain. Under mixotrophy-arabinose as well as mixotrophy-mixed sugar condition, the AraFGH-possessing A-Ut2 strain showed DW values similar to those shown by the A-Ut4 strain. On the other hand, the AraE-possessing A-Ut1 strain showed marginally lower DW values than those observed for the A-Ut4 strain at all time points. In the presence of arabinose and glucose, the A-Ut strains did not show two distinct growth phases (Figs. 4a, b and 5a, b; Additional File 1: Figure S1b, d, f, h).

#### Sugar uptake assays

Sugar uptake measurements were made alongside the biomass estimations under mixotrophy-arabinose and mixotrophy-mixed sugar conditions. Under the mixotrophy-arabinose condition, the WT and A-Tr strains that lacked the arabinose-specific catabolic gene set *araBAD* showed no change in the amount of arabinose present in the medium, indicating their inability to utilize the sugar (Additional File 1: Figure S1a, c, e, g). The A-Ut strains which harbored the *araBAD* gene set showed varying degrees of arabinose consumption. Under the mixotrophy-arabinose condition, the AraJ-possessing A-Ut3 strain showed arabinose uptake greater than those exhibited by the A-Ut1, A-Ut2 and A-Ut4 strains. At the 7th day of culture, the A-Ut3 strain showed ~ 243% greater arabinose uptake than the A-Ut4 strain (Fig. 4c, Additional File 1: S1b, d, f, h).

When grown under the mixotrophy-mixed sugar condition, the A-Ut3 strain showed arabinose consumption greater than the other A-Ut strains in accordance with the DW measurements. At the 7th day of culture, the A-Ut3 strain showed ~ 102% greater arabinose uptake than that by the A-Ut4 strain. Under mixotrophy, in the presence of arabinose alone as well as the mixed sugars, arabinose consumption exhibited by the A-Ut1 and A-Ut2 strains was found to be marginally lesser and similar to that by the A-Ut4 strain, respectively. The absence of two distinct growth phases and the simultaneous uptake of arabinose and glucose did not indicate diauxie in these strains, although glucose was consumed more efficiently than arabinose. (Fig. 5b–d; Additional File 1: Figure S1b, d, f, h). These results demonstrate that among the transporters under study, heterologous expression of only AraJ enhances arabinose transport in *Synechocystis*, which is further processed by the engineered isomerase catabolic pathway.

### Study of AraJ expression in AraE-possessing A-Ut strain

#### Construction of recombinant strain A-Ut5

To generate a strain co-expressing AraE and AraJ transporters, *araJ* was introduced into *araE*-possessing A-Ut1 strain. The resultant strain was denoted as A-Ut5 (Table 1).

#### Verification of genetic transformation and segregation of A-Ut5

**Fig. 6:**
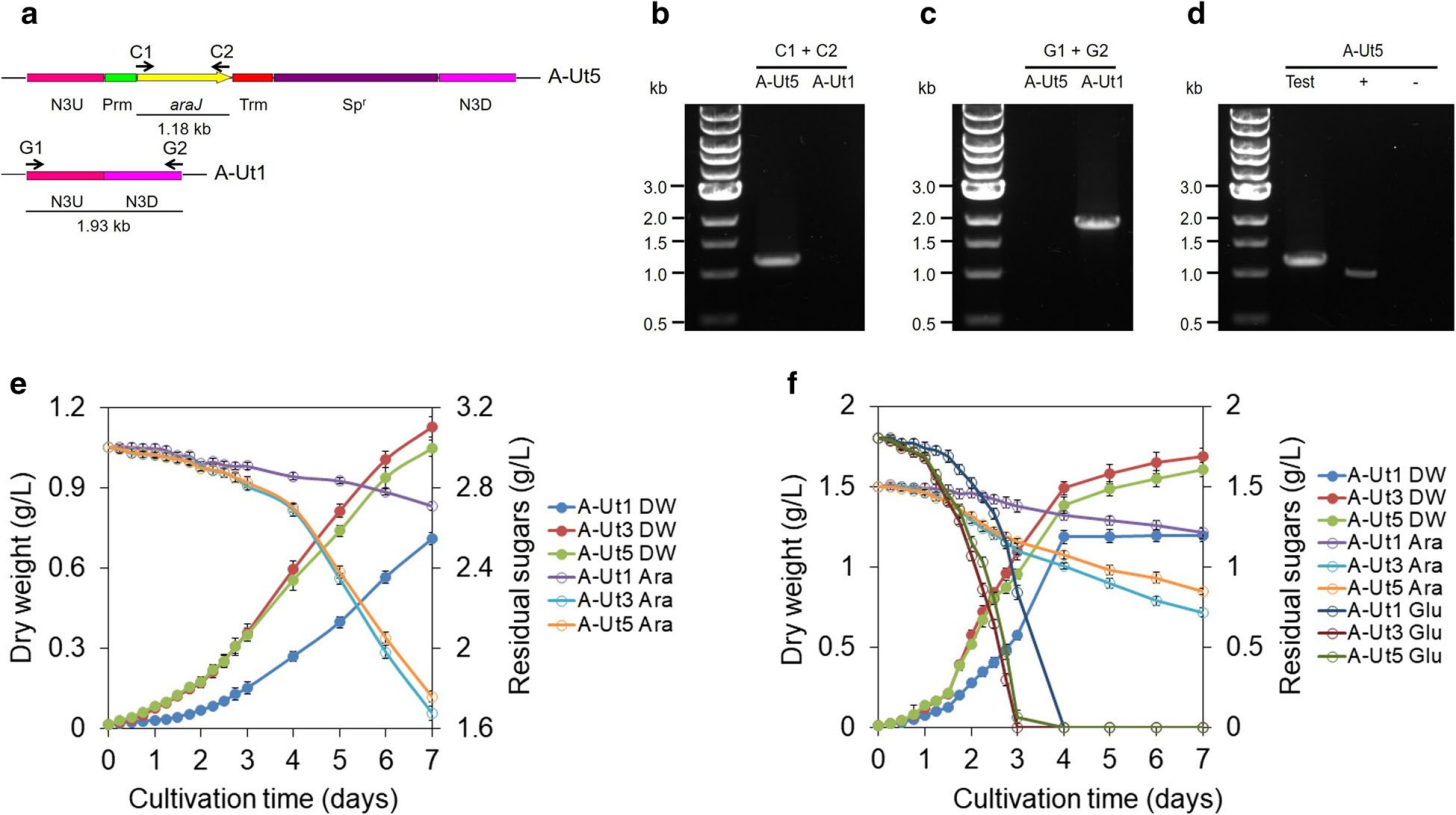
Construction of A-Ut5 strain and comparison of growth and sugar consumption between A-Ut1, A-Ut3 and A-Ut5. **a** *araJ* was inserted into neutral site 3 under the control of *psbA2* promoter (Prm) and 5ST1T2 terminator (Trm) in the A-Ut1 genome, using upstream (N3UP) and downstream (N3DW) regions for homologous recombination. The spectinomycin resistance cassette (Sp^r^) was used for selection and segregation of the transformant. Numbered letters and arrows represent the primer pairs and the direction of the amplification, respectively. **b** The presence of *araJ* was verified with the help of primers C1-C2, which generated a ~1.18 kb band. A-Ut1 strain served as the negative control. **c** Chromosomal segregation was examined with the help of primers G1-G2. In the absence of uninterrupted neutral site 3, the primers failed to generate a PCR product, whereas the A-Ut1 strain included as the positive control produced a ~1.93 kb band. **d** Transcription of *araJ* was demonstrated with the help of primers C1-C2, represented as ‘Test’, where the product of ~1.18 kb was obtained. In the positive control (+), *petA*-specific primers produced a ~0.99 kb band from cDNA template. In the negative control (-), primers C1-C2 failed to amplify *araJ* from pure RNA sample. **e** A-Ut1, A-Ut3 and A-Ut5 were grown under mixotrophy in the presence of 20 mM (3.00 g/L) L-arabinose. Dry biomass and residual sugars were measured in the cultures grown in triplicate. (f) A-Ut1, A-Ut3 and A-Ut5 were grown under mixotrophy in the presence of 10 mM (1.50 g/L) L-arabinose and 10 mM (1.80 g/L) D-glucose. Dry biomass and residual sugars were measured in the cultures grown in triplicate

To verify the presence of *araJ* in the recombinant strain generated after the third round of transformation, *araJ*-specific primers C1/C2 were used. The primer pair generated approximately 1.18 kb PCR product (Fig. 6a, b). To verify segregation of the recombinant, the forward primer used to amplify the upstream region and the reverse primer used to amplify the downstream region of neutral site 3 were used (denoted here as G1/G2) (Ranade et al. 2015). Extension times in the PCR cycles were set just sufficient to amplify the uninterrupted neutral site 3. PCR amplifications produced a ~ 1.93 kb band representing neutral site 3 only in the WT positive control. PCR did not generate any product in A-Ut5 indicating the absence of the uninterrupted neutral site and hence complete segregation of the recombinant strain (Fig. 6a, c).

#### Verification of *araJ* transcription in A-Ut5

After verification of segregation, transcription of *araJ* in the A-Ut5 strain was examined by RT-PCR as described above. In the presence of cDNA template obtained from the A-Ut5 strain, primers C1/C2 for *araJ* generated the expected PCR product. Negative control reaction performed using pure RNA template and C1/C2 primers failed to amplify *araJ*. Positive control reaction run using cDNA template showed amplification of *petA* (Fig. 6a, d). The results indicate successful *araJ* transcription and verify quality of the RNA sample.

#### Study of biomass accumulation by A-Ut5

To study the growth pattern exhibited by the A-Ut5 strain in comparison to the AraE-possessing A-Ut1 and AraJ-carrying A-Ut3 strains, the strains were cultured under mixotrophy-arabinose and mixotrophy-mixed sugar conditions. Under both the growth conditions, A-Ut5 showed higher biomass than A-Ut1, and comparable but marginally lower yields to those exhibited by the A-Ut3 strain. Under mixotrophy-arabinose, at day 7, the biomass obtained for A-Ut5 was ~ 48% greater than the biomass for A-Ut1, and ~ 7% lesser than that for the A-Ut3 strain. Under the mixotrophy-mixed sugar condition, at the 7th day of culture, A-Ut5 showed ~ 34% greater and ~ 5% lesser biomass than those obtained for the A-Ut1 and A-Ut3 strains, respectively (Fig. 6e, f).

#### Study of sugar uptake by A-Ut5

Sugar uptake measurements were made alongside the biomass estimations. Under mixotrophy-arabinose and mixotrophy-mixed sugar conditions, arabinose uptake by the A-Ut5 strain was greater than that by the A-Ut1 strain and comparable but marginally lower than that by the A-Ut3 strain. At the 7th day of culture, under mixotrophy-arabinose, the sugar uptake by the A-Ut5 strain was found to be ~ 326% greater and ~ 6% lesser than those exhibited by the A-Ut1 and A-Ut3 strains, respectively. Similarly, under the mixotrophy-mixed sugar condition, the arabinose consumption by the A-Ut5 strain was ~ 126% greater and ~ 16% lesser than the arabinose uptake by the A-Ut1 and A-Ut3 strains, respectively (Fig. 6e, f). The results demonstrate that the introduction of AraJ transporter was able to improve the performance of the A-Ut5 strain over its A-Ut1 parent.

## Discussion

We have constructed and compared the abilities of *Synechocystis* strains to utilize the second most common pentose sugar, arabinose by the expression of heterologous genes. *E*. *coli* genes involved in the metabolism of arabinose have been used to metabolically engineer several other bacterial and yeast species (Mohagheghi et al. 2002; Deanda et al. 1996; Kawaguchi et al. 2008; Sasaki et al. 2009; Cao et al. 2017; Young et al. 2010; Zhang et al. 1998b). Hence, as in our previously reported xylose work (Ranade et al. 2015), *E*. *coli* was chosen as the source organism for the arabinose-specific genes, which were expressed under the control of a native light-dependent promoter, *psbA2* (Mohamed and Jansson 1989; Lindberg et al; 2010). This promoter has been used for the expression of several heterologous genes (Zhou et al. 2014). Exclusion of *E*. *coli*-specific regulatory elements associated with the heterologous genes and the provision of continuous light exposure, ensured constant induction of *psbA2* promoter.

*Synechocystis* lacks specialized arabinose transporters. However, as observed in the *Synechocystis* strains engineered to utilize xylose (Ranade et al. 2015), expression of the catabolic enzymes AraBAD alone was sufficient for the utilization of arabinose (Figs. 4a–c and 5a–c; Additional File 1: Figure S1h), indicating the involvement of endogenous sugar transporter/s. Therefore, arabinose uptake exhibited by the A-Ut strains possessing heterologous transporters pertained to the activities of both endogenous and heterologous transporters. MFS-type glucose transporter GlcP (also referred to as Gtr) is the lone characterized sugar transporter identified in *Synechocystis* so far (Pao et al. 1998; Zhang et al. 1998a). We have compared the arabinose-utilizing abilities of the engineered *Synechocystis* strains relying on the native sugar transporter/s with or without one of the three heterologous arabinose transporters. With this strategy, by studying DW accumulation and sugar uptake, we have attempted to compare the performance of the heterologous transporters when expressed individually in *Synechocystis*.

Our results have indirectly demonstrated the expression of the catabolic gene set at the protein level (Figs. 4a–c and 5a–c; Additional File 1: Figure S1b, d, f, h), indicating that *E*. *coli* ribosomal binding sites (hereafter RBSs) of the *araBAD* operon were able to function in *Synechocystis*. We are unable to comment about the RBSs of the *araFGH* gene set at this time. Based on the biomass accumulation and the arabinose uptake results, AraFGH-possessing A-Ut2 strain failed to show any advantage over the A-Ut4 strain which lacked any non-native transporter (Figs. 4a–c and 5a–c, Additional File 1: Figure S1d, h). Expression of only one of the three selected heterologous transporters was able to boost arabinose utilization in the corresponding transformant. Among the A-Ut strains, AraJ-possessing A-Ut3 strain showed the most remarkable results. A-Ut2 and A-Ut4 showed an intermediate ability to utilize arabinose and were found to be slightly more efficient than the A-Ut1 strain (Figs. 4a–c and 5a–c; Additional File 1: Figure S1b, d, f, h). We were unable to determine if AraFGH was functionally active, owing to its inability to improve the uptake of arabinose.

Under mixotrophy, in the presence of 20 mM arabinose, and 10 mM each of arabinose and glucose, A-Ut1 showed the lowest biomass yield and arabinose uptake among the A-Ut strains. Under mixotrophy-arabinose condition, A-Tr1 showed biomass yield comparable to that obtained under autotrophy. When compared to the other strains included in the study, A-Tr1 and A-Ut1 did not show any notable growth impairment under autotrophy and mixotrophy-glucose condition as well (Figs. 4a–c and 5a–c; Additional File 1: Figure S1a–h). AraE and xylose transporter XylE show a high degree of homology with 141 identical amino acid residues, out of 472 and 491 amino acids, respectively (Maiden et al. 1987). XylE has recently been shown to function very efficiently in *Synechocystis* (Ranade et al. 2015). The performance of A-Ut1 strain could be due to one or more of the following factors, such as inability of AraBAD enzymes to efficiently process arabinose imported via AraE, stress caused by the expression of AraE and AraBAD at protein level and by the presence of L-arabinose. Accumulation of L-Ru5P, an L-arabinose pathway intermediate, has been known to exert harmful effects in *E*. *coli* cells (Englesberg et al. 1962). Insufficient strength of the RBSs in *araBAD* gene set could contribute to slower processing of L-Ru5P leading to slower growth of the A-Ut1 strain. However, in *S*. *elongatus* PCC 7942, expression of *E*. *coli araE* had failed to improve arabinose uptake (Cao et al. 2017). Hence, it is also possible that AraE may not be functionally active when expressed in *Synechocystis*.

*E*. *coli ydeA* has been shown to code for an MFS efflux transporter for L-arabinose and isopropylthio-β-galactoside (IPTG) (Carolé et al. 1999). With respect to cell growth, *ydeA* was found to be dispensable. Transcriptional activation of the gene was shown to lower intracellular concentration of arabinose which inhibits the expression of *araBAD* operon. Based on high homology between YdeA and AraJ, a role of AraJ in the export of non-metabolizable arabinose structural analogues was suggested (Bost et al. 1999). Earlier, AraJ was thought to be involved in the import or processing of arabinose polymers (Reeder and Schleif 1991). Similarly, with the help of a functional genomics tool, AraJ was grouped with other transporters such as B1657 (YdhP), YdeA, and YicM of *E*. *coli*, and YbcI, YdhL, YtbD, and YfhI of *Bacillus subtilis*, and their role in the transport of arabinose polymers was suggested (Lolkema and Slotboom 1998). However, the role of AraJ in the transport of arabinose polymers was deemed less likely, since none of the MFS proteins has been reported to transport substrates with molecular mass greater than 1,000 Da (Paulsen et al. 1998). 13 *E*. *coli* MFS transporters able to either import or export L-arabinose have been identified. Over-expression of a number of these transporter-coding genes including *ydeE* showed various degrees of toxicity to the *E*. *coli* cells. It was suggested that, L-Ru5P toxicity may explain the need of arabinose efflux, and the efflux proteins may target intermediates of arabinose metabolism (Koita and Rao 2012). Based on the sequence analyses, AraJ has been suggested to be a multi-pass inner membrane protein, and its topological and transmembrane domains have been identified (The UniProt Consortium 2017).

In comparison to all other strains included in this study, under mixotrophy-glucose, AraJ-possessing A-Tr3 and A-Ut3 strains showed slower growth. Similarly, under mixotrophy-arabinose, A-Tr3 showed some degree of growth impairment. However, A-Tr3 and A-Ut3 did not show any significant differential growth under autotrophic conditions (Additional File 1: Figure S1a–h). The results could be related to those obtained by Koita and Rao (2012) in *E*. *coli*, who had observed growth impairment due to over-expression of 5 out of 13 arabinose-related transporters. Although Fritz et al. (2014) had found AraJ not being responsible for rapid off-switching of arabinose system in *E*. *coli* cells, there were indications of the involvement of AraJ in the efflux of L-arabinose. When grown in the presence of a range of arabinose concentrations, A-Ut3 was able to tolerate arabinose concentration as high as 160 mM, however maximum DW values were obtained in the presence of 60 mM arabinose. In contrast, AraE-carrying A-Ut1 strain begins to show growth impairment even in the presence of 20 mM arabinose (Fig. 4a, b; Additional File 1: Figures S1b, f and S2). The advantage of AraJ-possessing A-Ut3 strain suggests that the transport of L-arabinose mediated by AraJ is efficient, or sufficient for the AraBAD enzymes to process. It will be interesting to investigate whether AraJ also carries out the efflux of L-arabinose, and if it can serve as a safety valve to reduce metabolic stress in a heterologous platform. The possible role of AraJ in the efflux of L-arabinose is depicted in Fig. 1.

To study whether the introduction of AraJ transporter can improve the performance of AraE-possessing A-Ut1 strain, A-Ut5 strain was generated. Under mixotrophy-arabinose and mixotrophy-mixed sugar conditions, the A-Ut5 strain showed higher DW and arabinose consumption than the A-Ut1 strain. The A-Ut5 strain’s performance was comparable to that exhibited by the A-Ut3 strain (Fig. 6e, f). We speculated that one of the reasons for the inability of A-Ut5 to show greater DW and arabinose utilization than A-Ut3 could be limitations in the ability of the AraBAD enzymes to funnel L-arabinose into PPP. In an attempt to improve the catabolic activity of AraBAD by means of modifying RBSs, we created two modified *araBAD* gene sets by replacing the IR regions with a part of the native *psbA2* promoter, CAAATACATAAGGAATTATAACCAA and a 12 bp long region of *E*. *coli* origin, AGGAGGTAATAT (Zurbriggen et al. 2012). Although a predictive design tool developed by Salis et al. (2009) had predicted advantage of the aforementioned RBSs over the original RBSs, the results indicated otherwise. The strains possessing both the modified gene sets showed decrease in DW yields (Additional File 1: Table S1, Fig. S3).

AraJ has been predicted to be a member of the drug H^+^ antiporter (DHA1) family (Bost et al. 1999; Kanehisa et al. 2016). Proton-motive force-driven symporters and antiporters have been shown to behave like uniporters in the presence of a strong concentration gradient of their substrates (Zhang et al. 2015). Although the exact mechanisms of AraJ activities have not yet been experimentally demonstrated, based on predictive models for protein function, available literature and our results, it is a possibility that AraJ is a proton antiporter which carries out the uptake of L-arabinose by facilitated diffusion.

The ability of AraJ to function as an L-arabinose transporter can be exploited for biotechnological applications. The arabinose-utilizing strains, especially A-Ut3 can be modified further to enhance its ability to grow on arabinose. As discussed previously, several approaches such as codon optimization of the arabinose-specific genes, increasing the strength of RBSs, laboratory evolution, exploration of genomes of other bacteria and algae for potential arabinose-specific genes can be followed. The arabinose-utilizing strain/s can be further engineered to metabolize additional carbon sources, such as xylose, and to delete glycogen synthase gene/s, to allow availability of more carbon for biotechnological conversion (Ranade et al. 2015). To the best of our knowledge, we are among one of the first groups to express L-arabinose catabolic pathway in cyanobacteria, and to show the role of AraJ as an arabinose transporter. Another significance of the work lies in the functional expression of AraJ in *Synechocystis*, even prior to the determination of its function in the host organism.

## Supplementary Information

**Additional file 1:Figure S1.** Growth and sugar consumption by the arabinose-specific strains under various conditions. Synechocystis strains (a) A-Tr1, (b) A-Ut1, (c) A-Tr2, (d) A-Ut2, (e) A-Tr3, (f) A-Ut3, (g) WT and (h) A-Ut4 were grown as three biological replicates, and the data are presented as means ± standard deviations. DW and residual sugars were estimated every 6 hours for the first 3 days followed by every 24 hours for the next 4 days. Blue line with filled squares, red lines with filled diamonds, light green line with filled triangles and violet line with filled circles represent growth under autotrophy, mixotrophy (20 mM arabinose), mixotrophy (20 mM glucose) and mixotrophy (10 mM each of arabinose and glucose) respectively. Similarly, light blue line with empty squares, orange line with empty circles and sky-blue line with crosses represent arabinose uptake under mixotrophy (20 mM arabinose), arabinose uptake under mixotrophy (10 mM each of arabinose and glucose) and glucose uptake under mixotrophy (10 mM each of arabinose and glucose) respectively. BG-11 media with 20 mM arabinose, 20 mM glucose, 10 mM arabinose and 10 mM glucose contain 3.0026, 3.6032, 1.5013 and 1.8016 g/L of the sugars respectively. **Figure S2.** Growth of A-Ut3 under various arabinose concentrations. A-Ut3 was grown in the presence of 0 mM (autotrophy) to160 mM arabinose as three biological replicates, and the data are presented as means ± standard deviations. DW was estimated every 24 hours for 7 days. Lines with filled circles represent growth in the presence of various arabinose concentrations, as specified within the figure. **Figure S3.** Growth of Synechocystis strains under mixotrophy in the presence of 20 mM arabinose. Synechocystis strains were grown in the presence of 20 mM (3.0026 g/L) L-arabinose. DW values estimated at the end of the seventh day are presented in the figure. Data were collected from three biological replicates and presented as means ± standard deviations. **Table S1.** Additional Synechocystis strains generated by modification of ribosome binding sites (RBSs) within araBAD gene set.

## Data Availability

All data generated or analyzed during this study are included in this published article and the additional file. The *Synechocystis* strains generated in this study can be obtained from the corresponding author with discretion upon reasonable request.

## Abbreviations

ABC: ATP binding cassette
DW: Dry weight
D-Xu5P: D-xylulose-5-phosphate
L-Ru5P: L-ribulose-5-phosphate
MFS: Major facilitator superfamily
RBS: Ribosomal binding sites
*Synechocystis*: *Synechocystis* sp. PCC 6803
WT: Wild-type strain
Mixotrophy-glucose: Mixotrophy with 20 mM glucose
Mixotrophy-arabinose: Mixotrophy with 20 mM arabinose
Mixotrophy-mixed sugar: Mixotrophy with 10 mM each of arabinose and glucose
