# Supplementary material for: Escherichia coli AraJ boosts utilization of arabinose in metabolically engineered cyanobacterium Synechocystis sp. PCC 6803
Source: AMB Express. 2021 Aug 13;11:115. doi: 10.1186/s13568-021-01277-7 (PMC8363721; doi:10.1186/s13568-021-01277-7)
Supplement: Supplementary file 1 — Additional file 1:Figure S1. Growth and sugar consumption by the arabinose-specific strains under various conditions. Synechocystis strains (a) A-Tr1, (b) A-Ut1, (c) A-Tr2, (d) A-Ut2, (e) A-Tr3, (f) A-Ut3, (g) WT and (h) A-Ut4 were grown as three biological replicates, and the data are presented as means ± standard deviations. DW and residual sugars were estimated every 6 hours for the first 3 days followed by every 24 hours for the next 4 days. Blue line with filled squares, red lines with filled diamonds, light green line with filled triangles and violet line with filled circles represent growth under autotrophy, mixotrophy (20 mM arabinose), mixotrophy (20 mM glucose) and mixotrophy (10 mM each of arabinose and glucose) respectively. Similarly, light blue line with empty squares, orange line with empty circles and sky-blue line with crosses represent arabinose uptake under mixotrophy (20 mM arabinose), arabinose uptake under mixotrophy (10 mM each of arabinose and glucose) and glucose uptake under mixotrophy (10 mM each of arabinose and glucose) respectively. BG-11 media with 20 mM arabinose, 20 mM glucose, 10 mM arabinose and 10 mM glucose contain 3.0026, 3.6032, 1.5013 and 1.8016 g/L of the sugars respectively. Figure S2. Growth of A-Ut3 under various arabinose concentrations. A-Ut3 was grown in the presence of 0 mM (autotrophy) to160 mM arabinose as three biological replicates, and the data are presented as means ± standard deviations. DW was estimated every 24 hours for 7 days. Lines with filled circles represent growth in the presence of various arabinose concentrations, as specified within the figure. Figure S3. Growth of Synechocystis strains under mixotrophy in the presence of 20 mM arabinose. Synechocystis strains were grown in the presence of 20 mM (3.0026 g/L) L-arabinose. DW values estimated at the end of the seventh day are presented in the figure. Data were collected from three biological replicates and presented as means ± standard deviations. Tabl [file 13568_2021_1277_MOESM1_ESM.pdf]

Supplementary Material

Escherichia coli AraJ plays a role in utilization of arabinose in metabolically engineered cyanobacterium *Synechocystis* sp. PCC 6803

Saurabh Ranade[1] and Qingfang He[1*]

Author Information

Affiliations

[1] Department of Biology, University of Arkansas at Little Rock, 2801 South University Avenue, Little Rock, AR 72204, USA

Corresponding Author

* Correspondence to Qingfang He, 2801 South University Avenue, ETAS 300Q, Little Rock AR 72204, Phone: 501-569-8033, Fax: 501-569-8020, Email: qfhe@ualr.edu

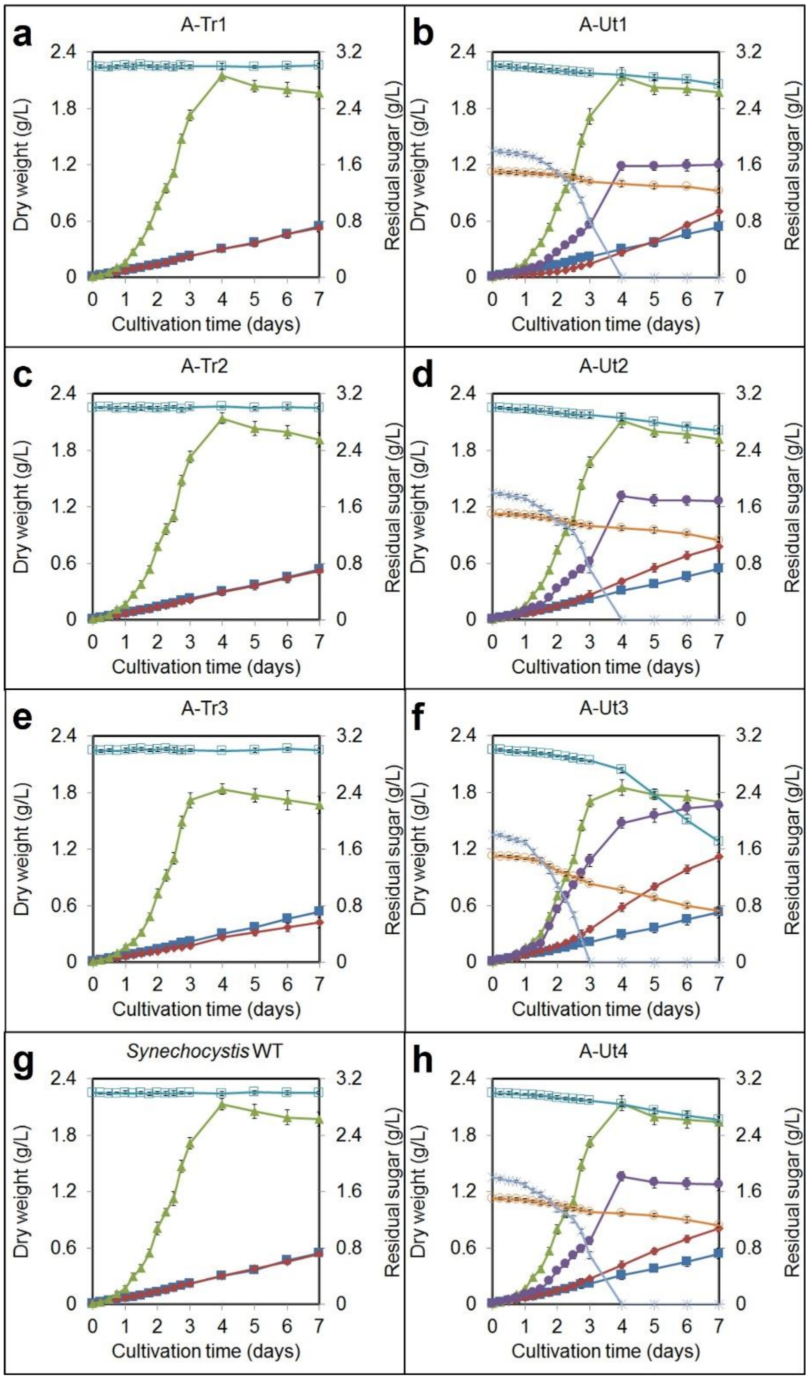

Fig. S1 Growth and sugar consumption by the arabinose-specific strains under various conditions

Synechocystis strains **(a)** A-Tr1, **(b)** A-Ut1, **(c)** A-Tr2, **(d)** A-Ut2, **(e)** A-Tr3, **(f)** A-Ut3, **(g)** WT and **(h)** A-Ut4 were grown as three biological replicates, and the data are presented as means ± standard deviations. DW and residual sugars were estimated every 6 hours for the first 3 days followed by every 24 hours for the next 4 days.

Blue line with filled squares, red lines with filled diamonds, light green line with filled triangles and violet line with filled circles represent growth under autotrophy, mixotrophy (20 mM arabinose), mixotrophy (20 mM glucose) and mixotrophy (10 mM each of arabinose and glucose) respectively. Similarly, light blue line with empty squares, orange line with empty circles and sky-blue line with crosses represent arabinose uptake under mixotrophy (20 mM arabinose), arabinose uptake under mixotrophy (10 mM each of arabinose and glucose) and glucose uptake under mixotrophy (10 mM each of arabinose and glucose) respectively. BG-11 media with 20 mM arabinose, 20 mM glucose, 10 mM arabinose and 10 mM glucose contain 3.0026, 3.6032, 1.5013 and 1.8016 g/L of the sugars respectively.

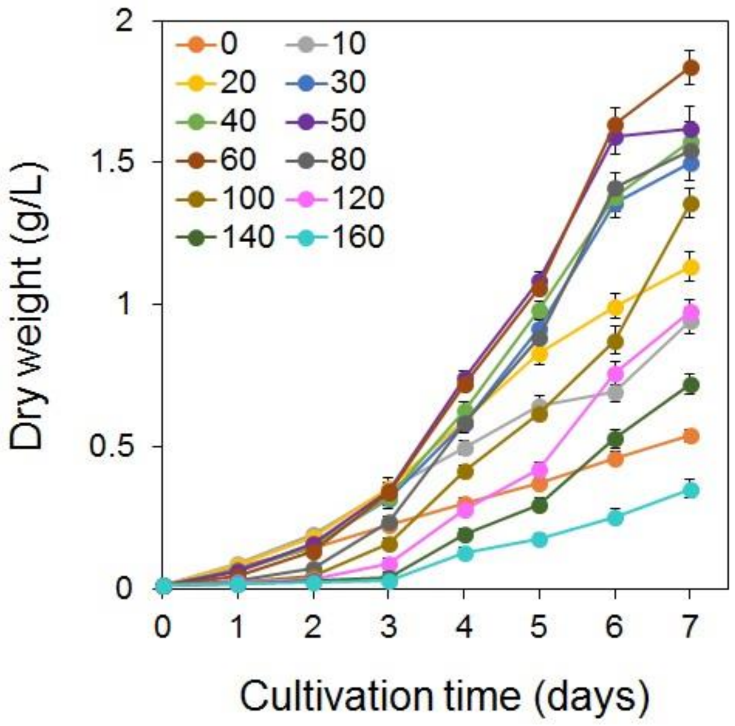

Fig. S2 Growth of A-Ut3 under various arabinose concentrations

A-Ut3 was grown in the presence of 0 mM (autotrophy) to160 mM arabinose as three biological replicates, and the data are presented as means ± standard deviations. DW was estimated every 24 hours for 7 days. Lines with filled circles represent growth in the presence of various arabinose concentrations, as specified within the figure.

Table S1 Additional *Synechocystis* strains generated by modification of ribosome binding sites (RBSs) within *araBAD* gene set.

Strain	Genotype	Description
A-Ut6	*araE*-Δ*Neu 1*::*araBAD* mod 1-Δ*Neu 3*	*araE* inserted at neutral site 1 (near *slr1285*), *araBAD* gene set mod 1 inserted at neutral site 3 (*slr1608*)
A-Ut7	*araFGH*-Δ*Neu 1*::*araBAD* mod 1-Δ*Neu 3*	*araFGH* gene set inserted at neutral site 1 (near *slr1285*), *araBAD* gene set mod 1 inserted at neutral site 3 (*slr1608*)
A-Ut8	*araJ*-Δ*Neu 1*::*araBAD* mod 1-Δ*Neu 3*	*araJ* gene inserted at neutral site 1 (near *slr1285*), *araBAD* gene set mod 1 inserted at neutral site 3 (*slr1608*)
A-Ut9	*araBAD* mod 1-Δ*Neu 3*	*araBAD* gene set mod 1 inserted at neutral site 3 (*slr1608*)
A-Ut10	*araE*-Δ*Neu 1*::*araBAD* mod 2-Δ*Neu 3*	*araE* inserted at neutral site 1 (near *slr1285*), *araBAD* gene set mod 2 inserted at neutral site 3 (*slr1608*)
A-Ut11	*araFGH*-Δ*Neu 1*::*araBAD* mod 2-Δ*Neu 3*	*araFGH* gene set inserted at neutral site 1 (near *slr1285*), *araBAD* gene set mod 2 inserted at neutral site 3 (*slr1608*)
A-Ut12	*araJ*-Δ*Neu 1*::*araBAD* mod 2-Δ*Neu 3*	*araJ* gene inserted at neutral site 1 (near *slr1285*), *araBAD* gene set mod 2 inserted at neutral site 3 (*slr1608*)
A-Ut13	*araBAD* mod 2-Δ*Neu 3*	*araBAD* gene set mod 2 inserted at neutral site 3 (*slr1608*)

araBAD mod 1 gene set was constructed by replacing original intergenic regions between *araB*-*araA* and *araA*-*araD* genes with *Synechocystis*-specific *psbA2* promoter's RBS-containing region, CAAATACATAAGGAATTATAACCAA. *araBAD* mod 2 gene set was constructed by replacing the intergenic regions between *araB*-*araA* and *araA*-*araD* genes with *E. coli*-specific RBS-containing region, AGGAGGTAATAT (Zurbriggen et al. 2012).

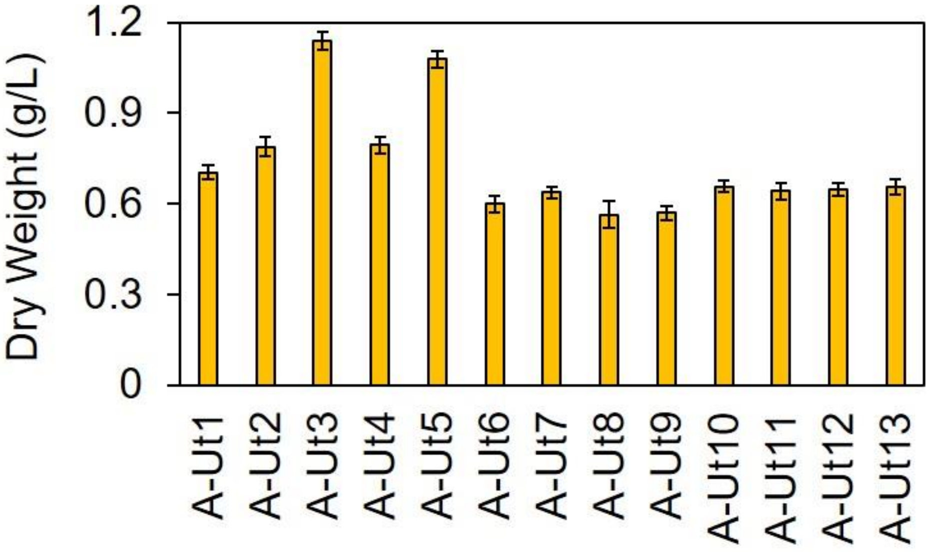

Fig. S3 Growth of *Synechocystis* strains under mixotrophy in the presence of 20 mM arabinose.

Synechocystis strains were grown in the presence of 20 mM (3.0026 g/L) L-arabinose. DW values estimated at the end of the seventh day are presented in the figure. Data were collected from three biological replicates and presented as means ± standard deviations.

References

Zurbriggen A, Kirst H, Melis A (2012) Isoprene production via the mevalonic acid pathway in *Escherichia coli* (Bacteria). Bioenergy Res 5:814-828. https://doi.org/10.1007/s12155-012-9192-4
